# Antimicrobial and antioxidant activity of *Evernia prunastri* extracts and their isolates

**DOI:** 10.1007/s11274-021-03099-y

**Published:** 2021-07-07

**Authors:** A. Shcherbakova, A. A. Strömstedt, U. Göransson, O. Gnezdilov, A. Turanov, D. Boldbaatar, D. Kochkin, G. Ulrich-Merzenich, A. Koptina

**Affiliations:** 1 Volga State University of Technology, Lenin Sq., 3, Yoshkar-Ola, Russia 424000; 2 Pharmacognosy, Department of Pharmaceutical Biosciences, Uppsala University, 751 24 Uppsala, Sweden; 3 FRC Kazan Scientific Center, Zavoisky Physical-Technical Institute, Russian Academy of Sciences, Sibirsky Tract, 10/7, Kazan, Russia 420029; 4 The Liver Center, Dalai Tower, Unesco Street 31, Sukhbaatar District, Ulaanbaatar, 14230 Mongolia; 5 Faculty of Biology, Lomonosov Moscow State University, GSP-1, 1-12 Leninskiye Gory, Moscow, Russia 119234; 6 Medical Clinic III, AG Synergy Research and Experimental Medicine, University Hospital Bonn, Venusberg-Campus 1, 53127 Bonn, Germany

**Keywords:** Antimicrobial activity, Anti-oxidative activity, *Evernia prunastri* (L.) Ach., Evernic acid, Lichen, Usnic acid

## Abstract

**Abstract:**

Lichens are symbiotic organisms formed by a fungus and one or more photosynthetic partners which are usually alga or cyanobacterium. Their diverse and scarcely studied metabolites facilitate adaptability to extreme living conditions. We investigated *Evernia prunastri* (L.) Ach., a widely distributed lichen, for its antimicrobial and antioxidant potential. *E. prunastri* was sequentially extracted by hexane (Hex), dichloromethane (DCM) and acetonitrile (ACN) that were screened for their antioxidant and antimicrobial (against *Staphylococcus aureus*, *Pseudomonas aeruginosa*, *Escherichia coli and Candida albicans*) activities. The Hex extract possessed the highest antioxidant capacity (87 mg ascorbic acid/g extract) corresponding to the highest content of phenols (73 mg gallic acid/g extract). The DCM and Hex extracts were both active against *S. aureus* (MICs of 4 and 21 µg/ml, respectively) but were less active against Gram-negative bacteria and yeast. The ACN extract exhibited activity on both *S. aureus* (MIC 14 µg/ml) and *C. albicans* (MIC 38 µg/ml) and was therefore further fractionated by silica gel column chromatography. The active compound of the most potent fraction was subsequently characterized by ^1^H and ^13^C-NMR spectroscopy and identified as evernic acid. Structural similarity analyses were performed between compounds from *E. prunastri* and known antibiotics from different classes. The structural similarity was not present. Antioxidant and antimicrobial activities of *E. prunastri* extracts originate from multiple chemical compounds; besides usnic acid, most notably evernic acid and derivatives thereof. Evernic acid and its derivatives represent possible candidates for a new class of antibiotics.

**Graphic abstract:**

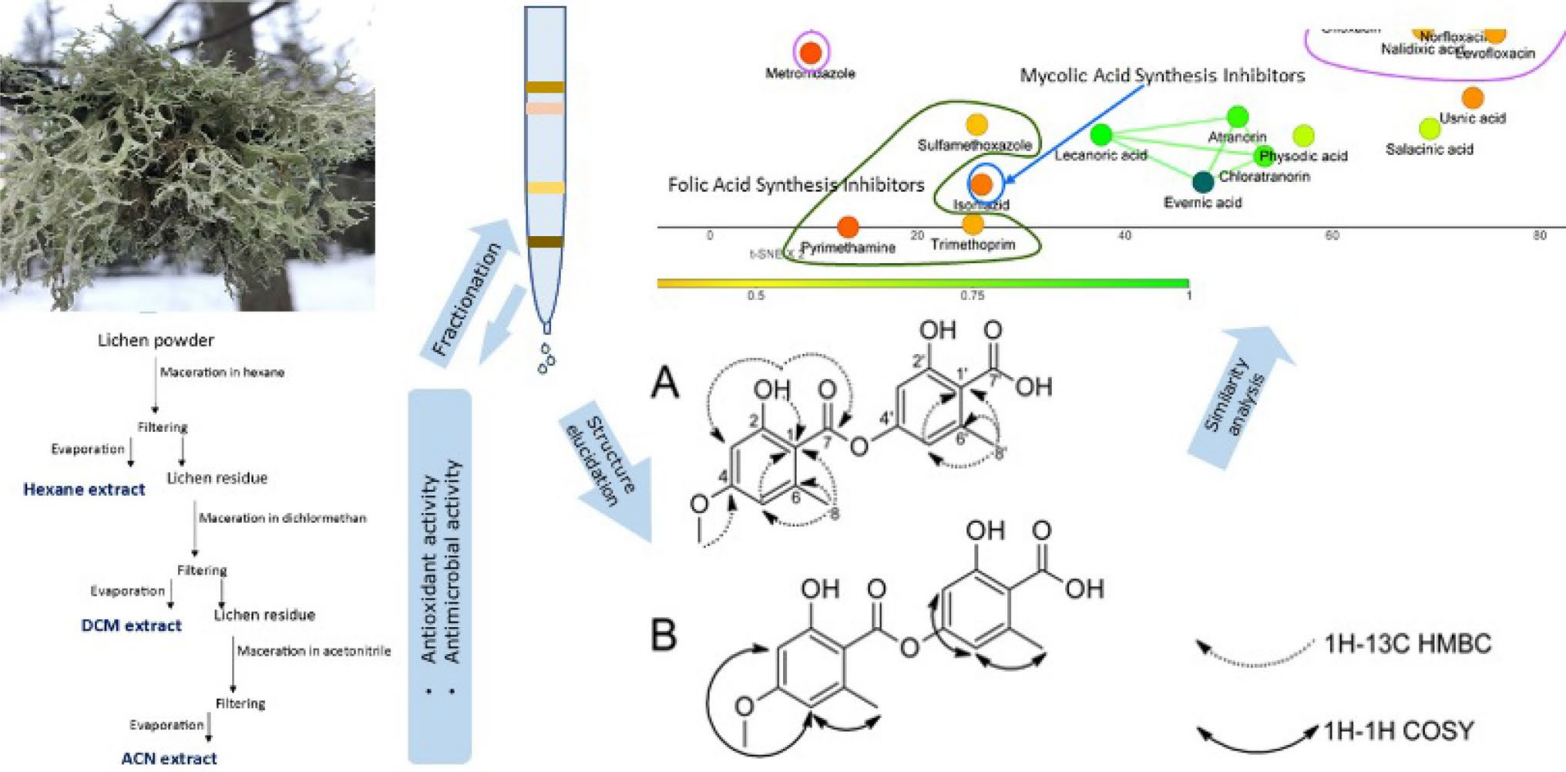

**Supplementary Information:**

The online version contains supplementary material available at 10.1007/s11274-021-03099-y.

## Introduction

Antibiotic resistance is one of the biggest threats to global health and development today that is not counterbalanced by the development of new therapeutic agents: no new classes of antibiotics have been developed since 1987 (WHO 2020; Durand et al. 2019; Hutchings et al. 2019). New resistance mechanisms are emerging and spreading globally, threatening our ability to treat common infectious diseases (WHO 2020). Considering that the most clinically relevant classes of antibiotics are derived from natural products (Hutchings et al. 2019), we identified lichens as a potential source of new antimicrobial compounds.

Lichens are organisms consisting of a fungus and a photosynthetic partner (cyanobacteria or algae) in a symbiotic relationship (Calcott et al. 2018). This unique symbiosis generates diverse and proprietary biochemical compositions of lichen compounds which offer a vast potential for the discovery of novel classes of antimicrobial substances (Mitrovic et al. 2011; Zambare and Christopher 2012). For example, depsides, depsidones and usnic acid derivatives are so far uniquely found in lichens (Stocker-Wörgötter et al. 2013).

In vitro studies suggest that lichen extracts may have a broad spectrum of activity against Gram-positive and -negative bacteria, fungi, and protozoan parasites (e.g., *Schistosoma mansoni, Leishmania* spp*., Toxoplasma gondii, Plasmodium berghei, Trypanosoma cruzi*) as well as antiviral activity (Schmeda-Hirschmann et al. 2008; Fritis et al. 2013; Lauinger et al. 2013; Luz et al. 2015; Ranković and Kosanić 2015; Si et al. 2016; Pastrana-Mena et al. 2016; Calcott et al. 2018; Araújo et al. 2019). Additionally to a prominent antimicrobial activity, lichen compounds were shown to possess analgesic, anticoagulant, anti-inflammatory, antipyretic, antiproliferative, wound healing, sun protecting (against UV irradiation) (Kohlhardt-Floehr et al. 2010; Nguyen 2013), and high antioxidative properties (Crawford 2015; Ranković and Kosanić 2015).

The present study focuses on the evaluation of antimicrobial and antioxidative activities of the lichen *Evernia prunastri* (L.) Ach., genus *Evernia*.

**Fig. 1:**
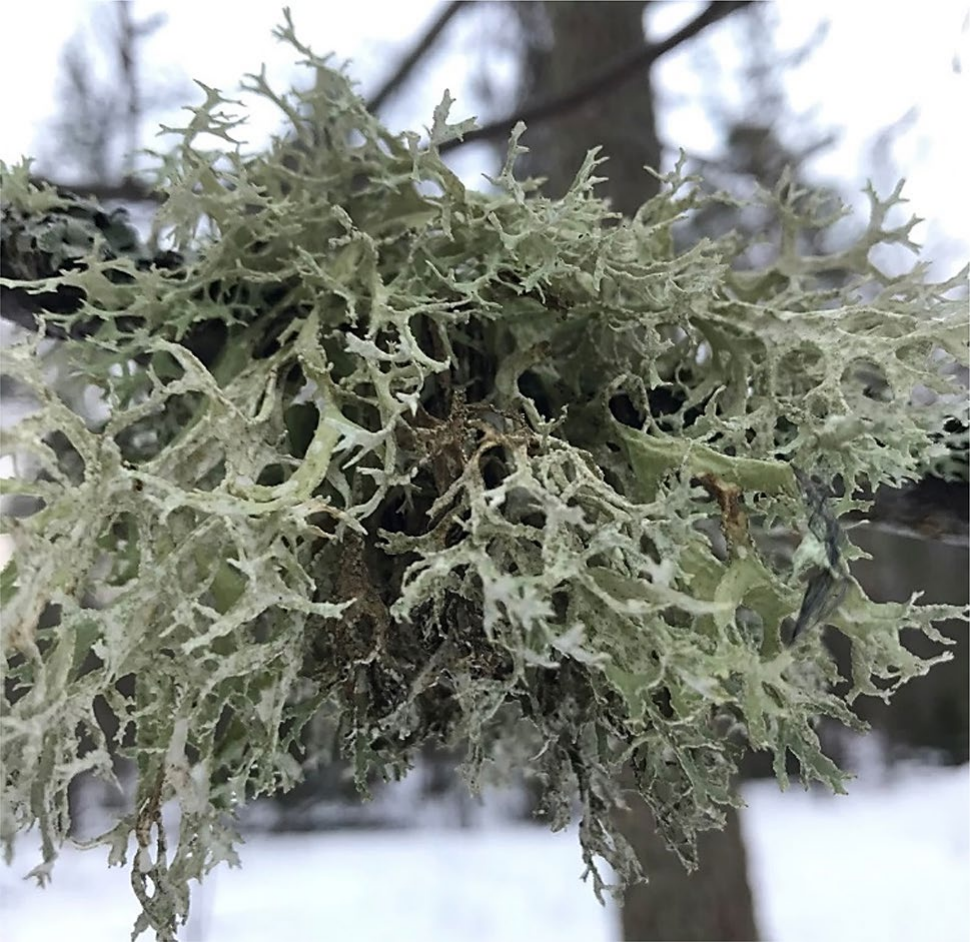
The lichen *Evernia prunastri* (L.) Ach.

*Evernia prunastri* is widely distributed across the Northern Hemisphere including Europe, North America and Asia. The thallus of *E. prunastri* (see Fig. 1) is on the upper side yellow-greenish, pale greenish, pale grey-green, with a cortex, on the underside predominantly whitish and somewhat channelled, without cortex, shrub-like, with granular-mealy soralia on the upper side and the margins, almost never with apothecium (disks usually pale yellowish, pale greenish or beige), without cylindrical isidia, usually up to 5 cm long. Lobes are 1–3 mm wide (Wirth and Anderegg 1995). It grows on deciduous trees, especially oaks and other broadleaf trees, or shrubs (only occasionally on conifers in areas with high humidity) (Golubkova et al. 1996).

The medicinal use of *E. prunastri* was already known in ancient Greece where it was used as a decoction or ointment for the treatment of diseases of the “womb”, against fatigue and as an astringent (Crawford 2015). In the European early modern era, it was used to treat intestinal weakness, fever and pulmonary afflictions (Crawford 2015). However, the form of preparation is unknown.

So far over 70 different metabolites have been identified and characterized in *E. prunastri* (Joulain and Tabacchi 2009). The most studied compounds are usnic and evernic acids, atranorin (Golubkova et al. 1996; Joulain and Tabacchi 2009; Staples et al. 2020), chloroatranorin (Avalos and Vicente 1987; Staples et al. 2020), atranol and chlorobutanol (Joulain and Tabacchi 2009; Uter et al. 2013; Mowitz et al. 2013; Andersen et al. 2015).

Especially usnic acid is known for its broad-spectrum antimicrobial activity. It is described to possess antibacterial properties against many Gram-positive bacteria (e.g., *Streptococcus* spp., *Pneumococcus* spp., *Bacillus* spp., *Enterococcus* spp., *Staphylococcus aureus* (including methicillin resistant), *Listeria monocytogenes*) (Garcia Rowe et al. 1999; Vijayakumar et al. 2000; Behera et al. 2005; Wang et al. 2006), some Gram-negative bacteria (e.g., *Proteus vulgaris*, *Salmonella typhimurium*) (Yılmaz et al. 2004), and mycobacteria *Mycobacterium tuberculosis *(at MIC of 32 μg/ml) (Ingólfsdóttir 2002)*.* Usnic acid is also described as an antifungal (e.g. *Candida* spp.) (Proksa et al. 1996; Pires et al. 2012; Nithyanand et al. 2015), antiprotozoal (e.g. *Leishmania* ssp., *Trypanosoma cruzi*, *Trichomonas vaginalis*, liver stage parasites of *Plasmodium berghei)* (Fournet et al. 1997; De Carvalho et al. 2005; Lauinger et al. 2013; Pastrana-Mena et al. 2016; Derici et al. 2018) and antiviral (e.g. human papilloma virus) (Piorkowski 1957; Yamamoto et al. 1995; Sokolov et al. 2012) agent. The sodium salt of usnic acid had been used as an antibiotic (under trade name Binan and Usno) against Gram-positive bacteria and mycobacteria in the USSR since 1955 (Belodubrovskaya et al. 2004). Furthermore, usnic acid can be found in various oral dietary supplements including Lipokinetix® marketed as a weight loss agent but that is now withdrawn due to the fulminant hepatotoxicity and acute liver failure (Han et al. 2004; Pramyothin et al. 2004; Neff et al. 2004; Arneborn et al. 2005; Hsu et al. 2005). Interestingly, there is a claim that in a clinical study to treat *Mycobacterium tuberculosis*, 30 patients received usnic acid tablets (90 mg/day or 1.5 mg/kg/day) for about 71 days, and in another study for bronchitis, 91 patients were treated with 30 mg usnic acid tablets/day. 10 days were found to be the appropriate therapy period (Luzina and Salakhutdinov 2018).

Evernic acid has been studied much less than usnic acid and is described to have antibacterial activity against, for example, *Bacillus mycoides, B. subtilis, Klebsiella pneumoniae and Candida albicans* at MIC 0.25 mg/ml, against *Escherichia coli* at MIC 0.5 mg/ml and against *Aspergillus flavus, A. fumigatus, Penicillium purpurescens* and *P. verrucosum* at MIC 1 mg/ml (Kosanić et al., 2013) as well as against *Pseudomonas* *aeruginosa* quorum-sensing systems (Gökalsın and Sesal 2016). Some studies also suggest its effectiveness against *S. aureus* which could, however, not be confirmed in another study (Lauinger et al. 2013). Also, evernic acid is shown to have moderate activity on the Epstein-Barr virus activation inhibition (Yamamoto et al. 1995). Atranorin, among other compounds, has also been described to possess antibacterial and antiprotozoal activity (Araújo et al. 2015; Studzinska-Sroka et al. 2017).

Some lichen compounds (e.g., usnic acid, evernic acid, atranorin, fumarprotocetraric acid, lecanoric acid, diffractaic acid, lobaric acid, stictic acid, salazinic acid, physodic acid, psoromic acid, norstictic acid, protocetraric acid) are described to possess antioxidant properties (White et al. 2014; Araújo et al. 2015; Fernández-Moriano et al. 2017; Studzinska-Sroka et al. 2017).

In the current work, we investigated the antimicrobial and antioxidative properties of different extracts from the lichen *Evernia prunastri* (L.) Ach. and subsequently performed a bioassay-guided fractionation of the most active extract in order to isolate the active antimicrobial compound(s).

## Materials and methods

### Materials

Lichen samples of *Evernia prunastri* (L.) Ach. were collected in the Mari El Republic of the Russian Federation in June 2012. The lichen was identified by lichenologist G.A. Bogdanov of the Bolshaya Kokshaga Natural Reserve. The voucher specimen of the lichen was deposited at the Department of Forestry and Ecology, Volga State University of Technology, Yoshkar-Ola, Russia.

Antimicrobial assays were conducted using four clinical strains of human pathogens: *Escherichia coli* ATCC 25922, *Staphylococcus aureus* ATCC 29213, *Pseudomonas aeruginosa* ATCC 27853 and the yeast *Candida albicans* ATCC 90028. Tryptic Soy Broth (TSB) for *S. aureus*, *P. aeruginosa* and *C. albicans* cultivation and Luria Broth medium (LB) (Merck KGaA, Darmstadt, Germany) for *E. coli* cultivation were used.

### Sample preparation

#### Extraction

Air-dried powdered thalli of the lichens were extracted by sequential maceration with hexane, dichloromethane (DCM) and 60% acetonitrile in water (ACN) at room temperature for 24 h with each solvent. The extracts were filtered and then concentrated under reduced pressure in a rotary evaporator Rotavapor R (Buchi Labortechnik AG, Flawil, Switzerland). The dry extracts were stored at room temperature until usage.

#### Fractionation and isolation of chemical constituents

Chemical constituents of 60% ACN extract of *E. prunastri* were separated by silica gel column chromatography. The glass column with inner diameter of 40 mm was packed with silica gel 60 (Merck, Darmstadt, Germany). Four eluents were used sequentially: (1) cyclohexane: acetone (7:4, v/v); (2) cyclohexane: acetone: methanol (7:4:1, v/v/v); (3) cyclohexane: acetone: methanol (7:4:2, v/v/v); (4) methanol.

In total 91 fractions with volume of 8 ml each at flow rate of 15 ml/min were collected. Based on TLC analysis fractions containing identical compounds were combined. Batch 85 and 86–89 were further separated by semi-preparative and preparative RP-HPLC.

TLC was carried out on Silica gel 60 F254 0.25 mm Aluminium plates (Merck, Darmstadt, Germany). Solvent system cyclohexane: acetone: methanol (7:4:2, v/v/v) was used. Zones were visualized under UV light and by dipping into a vanillin-sulfuric acid reagent followed by heating at 120 °C.

Preparative and semi-preparative HPLC was conducted on Äkta Basic 10 HPLC system (Amersham Pharmacia Biotech, Sweden) with C18 columns. The flow rate was set to 10 and 4 ml/min and the UV-900 detector was operated at wavelengths of 215, 254 and 280 nm. The mobile phases consisted of 10% (A) and 60% (B) aqueous acetonitrile, both containing 0.05% trifluoroacetic acid. The gradient was set to 10–100% (B) over 21 min.

#### Characterization and identification of compounds

^1^H and ^13^C NMR spectra were recorded on a “Bruker AVANCE 400” of the Centre of Collective Facilities, FRC Kazan Scientific Centre, at operating frequencies of 400.13 and 100.62 MHz, respectively. Chemical shifts were measured with reference to the residual protons of the solvent (CDCl_3_, ^1^H, 7.26 ppm, ^13^C, 77.16 ppm).

### Antioxidant activity

#### Total antioxidant capacity

The total antioxidant activity of the studied extracts was evaluated by the phosphomolybdenum method as described by Manojlovich et al. (2012). Briefly, 0.2 ml of sample extract was combined with 3 ml of reagent solution (0.6 M sulfuric acid, 28 mM sodium phosphate and 4 mM ammonium molybdate). The tubes containing the reaction solution were incubated at 95 °C for 90 min. Then the absorbance of the solution was measured at 695 nm using a spectrophotometer against blank after cooling to room temperature. Methanol (0.3 ml) was used as the blank. Ascorbic acid (AA) was used as a standard and the total antioxidant capacity is expressed as milligrams of ascorbic acid per gram of the dry extract.

#### Total phenolic compounds

The total phenolic content was determined using the Folin–Ciocalteau (FC) method (Manojlovic et al. 2012). Shortly, extracts were diluted to the concentration of 1 mg/ml and aliquots of 0.5 ml were mixed with 2.5 ml of FC reagent (previously diluted ten-fold with distilled water) and 2 ml of NaHCO_3_ (7.5%). After 15 min of staying at 45 °C, the absorbance was measured at 765 nm on a spectrophotometer versus a blank sample. Total phenols were determined as gallic acid equivalents (mg GA/g extract), and the values are presented as means of triplicate analyses.

### Antimicrobial activity

#### Broth microdilution assay to measure minimal inhibitory concentration (MIC)

The Minimal Inhibitory Concentration (MIC) was determined for the extracts and fractions by a broth microdilution method previously described (Wiegand et al. 2008). Shortly, bacteria grown overnight in 3% TSB were rinsed with Tris buffer and diluted in refined LB to obtain a concentration of approximately 10^6^ CFU per ml as determined by OD600. The fractions were dissolved in 5% DMSO and a concentration range from 0.1 to 500 µg/ml was tested in a 96 well microplate. Ninety (90) µl of the bacterial suspension was administered into each well. The microplate was incubated at 37 °C for 6 h with *E. coli*, 8 h with *P. aeruginosa*, 9 h with *S. aureus*, 12 h with *C. albicans*. The incubation times for the different strains were chosen according to growth rate rendering approximate similarity in total mass, i.e., pellets of 2 mm in diameter (Strömstedt et al. 2017). The effects of all extracts were compared with the effect of usnic acid (the most studied antibiotic compound in lichens). Effects of the 5% DMSO solvent alone was also examined.

Experiments were performed at least in three separate replicates and were found to be consistent within each dilution factor. The MIC was taken as the concentration at which no visible pellet was observed.

### Computing the activities of the compounds from *E*. *prunastri*

#### Similarity/activity Cliffs analysis

Compounds of the *E. prunastri* extract (Staples et al. 2020) were structurally compared with main drugs of different antibiotics classes. The comparative study and visualization were performed using the DataWarrior software (5.2.1) (Sander et al. 2015). The Analyse Similarity/Activity Cliffs tool was applied to calculate and plot similarity (Guha and Van Drie 2008). Additionally, to visualize the chemical space of molecules, t-SNE (Stochastic Neighbour Embedding) visualization was performed (Van Der Maaten 2014; Karlov et al. 2019). This was followed by a synchronization of the similarity plot with t-SNE.

Similarity score of usnic acid and evernic acid in comparison to the antibiotics was calculated in DataWarrior software based on the comparison of descriptors—FragFp (Sander et al. 2015).

#### Prediction of toxicity risks

Prediction of four toxicity risks (Mutagenic, Tumorigenic, Reproductive Effective and Irritant) were estimated in the DataWarrior software (5.2.1) using the ‘Calculate properties’ tool (Sander et al. 2015). The analyses are based on fragment-based toxicity estimations compiled in the *Registry of Toxic Effect of Chemical Substances database* and the *World Drug Index database* (von Korff and Sander 2006).

### Statistical analyses

Statistical analyses were performed using Excel and Origin software packages. To determine the statistical significance of the antioxidant activity student’s t-test was utilized. All values are expressed as mean ± SD of three parallel measurements.

## Results

### Preparation of extracts and fractionation

The hexane, DCM and ACN macerations of the *Evernia prunastri* lichen yielded 137 mg (2.73% of sample dry weight), 190 mg (3.80% of sample dry weight) and 311 mg (6.22% of sample dry weight) of extracts, respectively.

The 60% ACN fraction was further fractionated to investigate subfractions and isolate and elucidate single active compounds. In total 91 fractions of the ACN extract of *E. prunastri* were collected. Based on the TLC analyses, fractions containing identical compounds were combined which resulted in 15 different solutions that were further used for the MIC assays.

### Antimicrobial activity

The inhibitory activity of different extracts of *E. prunastri* as well as of the fractions of the ACN extract was evaluated against clinical strains of four human pathogens, i.e., Gram-positive bacterium *Staphylococcus aureus,* Gram-negative bacteria *Pseudomonas aeruginosa* and *Escherichia coli*, and the fungus *Candida albicans.* Usnic acid, the lichen metabolite known for its antimicrobial properties, was used as a reference. The obtained results are presented in Tables 1 and 2.

**Table 1:** Antimicrobial activity of different extracts of the lichen *E. prunastri* and usnic acid

Samples	Minimum inhibitory concentration (MIC), µg/ml^‡^			
	*Staphylococcus aureus*	*Pseudomonas aeruginosa*	*Escherichia coli*	*Candida albicans*
*E. prunastri* (Hexane)	21 ± 9^(0.9)^	150 ± 41^(0.8)^	> 500	150 ± 50^(0.2)^
*E. prunastri* (DCM)	4 ± 1*	167 ± 33^(0.5)^	500 ± 1*	150 ± 50^(0.2)^
*E. prunastri* (60% ACN)	14 ± 3^(0.1)^	133 ± 33^(1.0)^	250 ± 1^(0.3)^	38 ± 13*
Usnic acid	21 ± 9	133 ± 33	225 ± 25	100 ± 1

^‡^Data expressed as a mean ± SD (n = 3 for each experiment)

*p < 0.01 vs. usnic acid

**Table 2:** Minimum inhibitory concentration (MIC) of fractions (see Supplementary Figure S1) obtained from acetonitrile: water (3:2) extract of *Evernia prunastri*

Fraction	Yield (mg)	MIC, µg/ml			
		*Staphylococcus aureus*	*Pseudomonas aeruginosa*	*Escherichia coli*	*Candida albicans*
I	5	> 500	> 500	250	500
II	3.4	> 500	> 500	> 500	> 500
III	12.4	62.5	> 500	> 500	> 500
IV	10.7	> 500	> 500	500	> 500
V	10.6	**1**.**95**	**31**.**25**	**31**.**25**	62.5
VI	31.8	**0**.**98**	**31**.**25**	**125**	62.5
VII	31.1	**1**.**95**	> 500	> 500	**31**.**25**
VIII	44.9	**0**.**49**	> 500	> 500	62.5
IX	12.6	3.91	> 500	> 500	125
X	13.0*	> 500	125	500	> 500
XI		500	125	500	> 500
XII		500	500	> 500	> 500
XIII		125	500	> 500	> 500
XIV	11.8	15.63	> 500	> 500	500
XV	224.6	125	125	500	500

*Fraction after #IX (with mass 13 mg) was further separated using RP-HPLC into 4 more fractions

Numbers in bold indicate susceptibility or intermediate antibacterial activity

Usnic acid was active against *S. aureus*, with a minimum inhibitory concentration (MIC) of 21 µg/ml. A high and significant effect was found for the *E. prunastri* DCM extract with a MIC of 4 µg/ml. For the *E. prunastri* Hexane extract, the ability to inhibit *S. aureus* was similar to the one of usnic acid. The *E. prunastri* ACN extract showed a MIC of 14 μg/ml, a slightly higher potency than usnic acid.

Against *P. aeruginosa,* the effect of usnic acid was comparable to the *E. prunastri* extracts and the measured MIC value of 133 µg/ml. Our estimated MIC values for the *E. prunastri* ACN, Hexane and DCM extracts (MIC values of 133, 150 and 167 µg/ml, respectively) were in the range with the one of usnic acid.

Against the Gram-negative bacteria *E. coli*, usnic acid was not effective (MIC value of 225 µg/ml). The MIC values of the *E. prunastri* ACN extract (250 µg/ml) was comparable to the one of usnic acid. The other two *E. prunastri* extracts showed even higher MIC values (Table 1).

Against *C. albicans,* the highest activity was seen with the *E. prunastri* ACN extract with a MIC value of 38 µg/ml. The effect of the extract is significantly higher than the effect of usnic acid with a MIC of 100 µg/ml. The *E. prunastri* Hexane and DCM extracts showed a low activity with a MIC of 150 µg/ml.

The *E. prunastri* ACN extract showed antimicrobial activity against all tested microorganisms and was therefore fractionated using column chromatography and HPLC into 15 fractions (Supplementary Figure S1) as described in the method section. The individual fractions were subsequently tested for their antimicrobial activity and the resulting MIC values are shown in Table 2.

Fractions V and VI showed high activities against all tested microorganisms. Comparably high activities were also seen for the fractions VII and VIII, but only against *S. aureus* and *C. albicans*. A reason for such similarity in antimicrobial activity may be explained by the cross-presence of one or more compound(s) in the neighbouring fractions.

Fraction VIII showed the highest activity against *S. aureus* with a MIC value of 2 μg/ml. Fraction VI had a slightly lower effect with a MIC of 4 μg/ml. This MIC is, however, still lower than the one of usnic acid (21 μg/ml) measured by the same method.

The activities of these fractions against Gram-negative bacteria are quite similar. The MIC values of fractions V and VI against *P. aeruginosa* were similar and significantly lower than the MIC value of usnic acid (p-value = 0.02). The activity of fraction V against *E. coli* is similar to the activity of this fraction against *P. aeruginosa.* In contrast, fraction VI is less effective against *E. coli*.

The effects of the fractions V, VI and VIII against *C. albicans* are the same and the effect of fraction VII is insignificantly higher (p-value = 0.34). All these fractions are more effective than usnic acid (p-value < 0.05).

In general, the antimicrobial activity of the fractions is higher than the one of the ACN extract itself.

Since the fractions V and VI inhibited the growth of all tested microorganisms, and, besides that, fraction VI showed a higher effect against *S. aureus*, it was chosen for further characterisation and NMR analysis.

### Identification of chemical compounds

Based on ^1^H and ^13^C NMR data, the primary compound of fraction VI was identified as evernic acid. Chemical shifts (δ, ppm) and coupling constants (J, Hz) for the extract and evernic acid standard are as follow:

#### Extract

^1^H NMR (CDCl3), δ, ppm (J, Hz): 2.59 s (3H, 8CH3), 2.76 s (3H, 8´CH3), 3.89 s (3H, OCH3), 6.42 m (2H, 3CH, 5CH), 6.57 d (1H, 5′CH3, J 2.0), 6.69 d (1H, 3′CH3, J 2.0), 11.08 s (3H, 2COH, 2′COH, 7′COH).

^13^C NMR (CDCl3), δ, ppm: 24.43, (8C), 24.73 (8′C), 55.57 (OCH3), 99.04 (3C), 104.38 (1C), 109.09 (1′C), 109.18 (3′C), 112.11 (5C), 117.09 (5′C), 143.59 (6C), 144.96 (6′C), 155.08 (4′C), 160.09 (2′C), 165.04 (2C), 165.47 (4C), 166.67 (7C), 169.70 (7´C).

#### Evernic acid

^1^H NMR (CDCl3), δ, ppm (J, Hz): 2.58 s (3H, 8CH3), 2.70 s (3H, 8′CH3), 3.88 s (3H, OCH3), 6.41 m (2H, 3CH, 5CH), 6.56 d (1H, 5′CH3, J 2.0), 6.68 d (1H, 3′CH3, J 2.0), 11.04 s (2H, 2COH, 2´COH), 11.41 s (1H, 7′COH).

^13^C NMR (CDCl3), δ, ppm: 24.24 (8C), 25.83 (8′C), 57.42 (OCH3), 98.78 (3C), 105.69 (1C), 109.06 (1′C), 109.13 (3′C), 114.58 (5C), 119.05 (5′C), 143.22 (6C), 144.75 (6′C), 155.08 (4′C), 160.24 (2′C), 164.00 (2C), 165.44 (4C), 166.66 (7C), 169.71 (7′C).

**Fig. 2:**
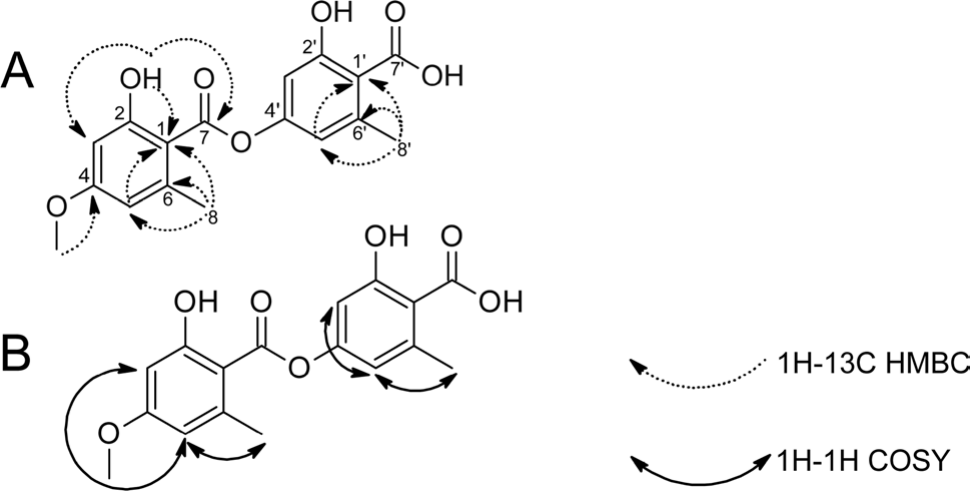
Structure and key ^1^H–^13^C HMBC (**A**) and ^1^H–^1^H COSY (**B**) correlations of the major compound from the fraction VI

The numbering of carbons is standard for depsides and depsidones, C-7 and C-8 refer to the carbons of the carboxylic acid and the methyl group, respectively (Narui et al. 1998). ^1^H–^13^C HMBC and ^1^H–^1^H COSY correlations were reasonable for the structure (Fig. 2).

The determined molecular formula and the NMR data analysis were compared to published literature data which confirms that the investigated compound is evernic acid ((2-hydroxy-4-[(2-hydroxy-4-methoxy-6-methylbenzoyl)oxy]-6-methylbenzoate) (Huneck and Yoshimura 1996; Narui et al. 1998). To further verify this, the NMR analysis of pure evernic acid was performed and its NMR spectra were compared with the NMR spectra of the ACN extract fraction VI. The spectra were similar enough to identify the primary active compound in the extract as evernic acid.

### Hypothetical mechanism of antimicrobial action

**Fig. 3:**
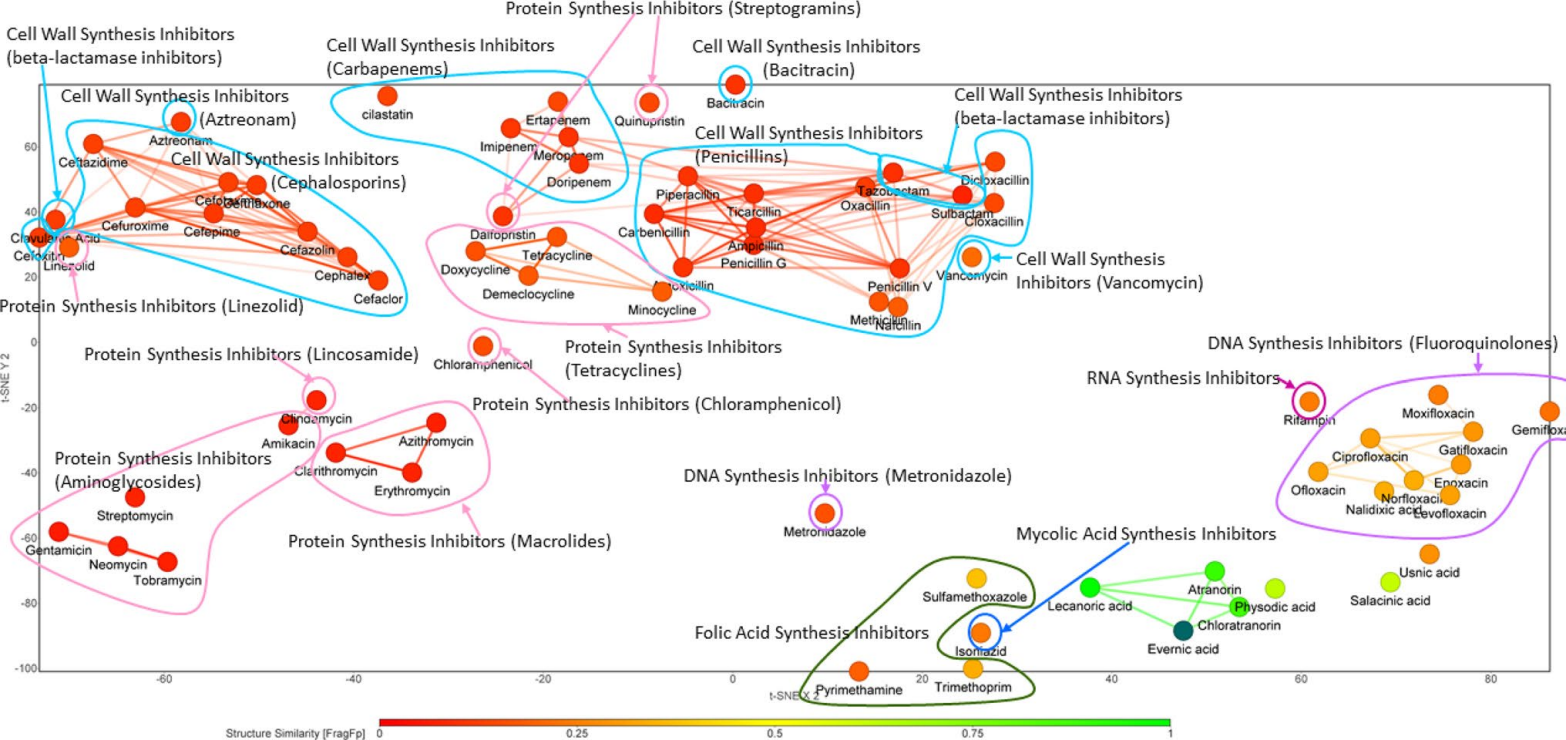
Similarity Chart of evernic acid vs. known classes of antibiotics with application t-SNE visualization approach. The colours from red to green indicate the range from no structural similarity to 100% similarity, respectively (Van Der Maaten 2014). The sample disposition depends on the similarity and is distributed according to sampling (Karlov et al. 2019). Structurally similar to evernic acid are the lichen compounds lecanoric acid, atranorin, and chloroatranorin (in green) as well as physodic acid, and salacinic acid (light green)

To elucidate further the potential mode of action of evernic acid, we analysed the structural similarity to the known antibiotic classes and its similarity to other lichen compounds. Interestingly evernic acid is structurally different from almost all main classes of antibiotics, with a slight similarity to folic acid synthesis inhibitors (mostly due to the ring system) (Fig. 3). However, evernic acid has a similarity with other lichen compounds as shown in Fig. 3. It is highly similar to lecanoric acid, atranorin and chloratronin, moderately similar to physodic acid and salacinic acid and less similar to usnic acid.

Usnic acid has more similarity to known antibiotics than evernic acid. The highest similarity is with a cell wall synthesis inhibitor — vancomycin (FragFp = 0.54) and RNA synthesis inhibitor — rifampin (FragFp = 0.52) (Supplementary Figure S2).

Evernic acid is moderately similar to folic acid synthesis inhibitors as sulfamethoxazole (FragFp = 0.38) and trimethoprim (FragFp = 0.34) (Supplementary Figure S2).

Both evernic acid and usnic acid showed quite identical similarity score to fluoroquinolones. Nalidixic acid and norfloxacin are the most similar compounds to evernic acid with FragFps of 0.34 and 0.33, respectively. Usnic acid possesses the highest similarity with moxifloxacin (FragFp = 0.51) and gatifloxacin (FragFp = 0.50).

### Possible toxicity of the *E*. *prunastri* extracts

The toxicity risks of the described compounds from *E. prunastri* were predicted using the DataWarrior software (von Korff and Sander 2006; Sander et al. 2015). Results are shown in Table 3. According to the prediction, none of the depsides associated with a toxicity risk. However, depsidones and usnic acid present toxicity risks, either as an irritant — physodic acid or through reproductive effects — salacinic acid and usnic acid (Table 3). None of the compounds was predicted to carry mutagenic or tumorigenic risks.

**Table 3:** Predicted toxicity risks for *E. prunastri* constituents based on fragment-based toxicity estimation compilated from Registry of Toxic Effect of Chemical Substances database and World Drug Index database (von Korff and Sander 2006)

Name	Mutagenic	Tumorigenic	Reproductive effective	Irritant
Physodic acid	None	None	None	High
Chloratranorin	None	None	None	None
Salacinic acid	None	None	High	None
Atranorin	None	None	None	None
Usnic acid	None	None	High	None
Evernic acid	None	None	None	None
Lecanoric acid	None	None	None	None

### Antioxidative properties of the *E*. *prunastri* extracts

In this study, three lichen *E. prunastri* extracts (Hexane, DCM, ACN) were investigated for their antioxidant potential.

**Fig. 4:**
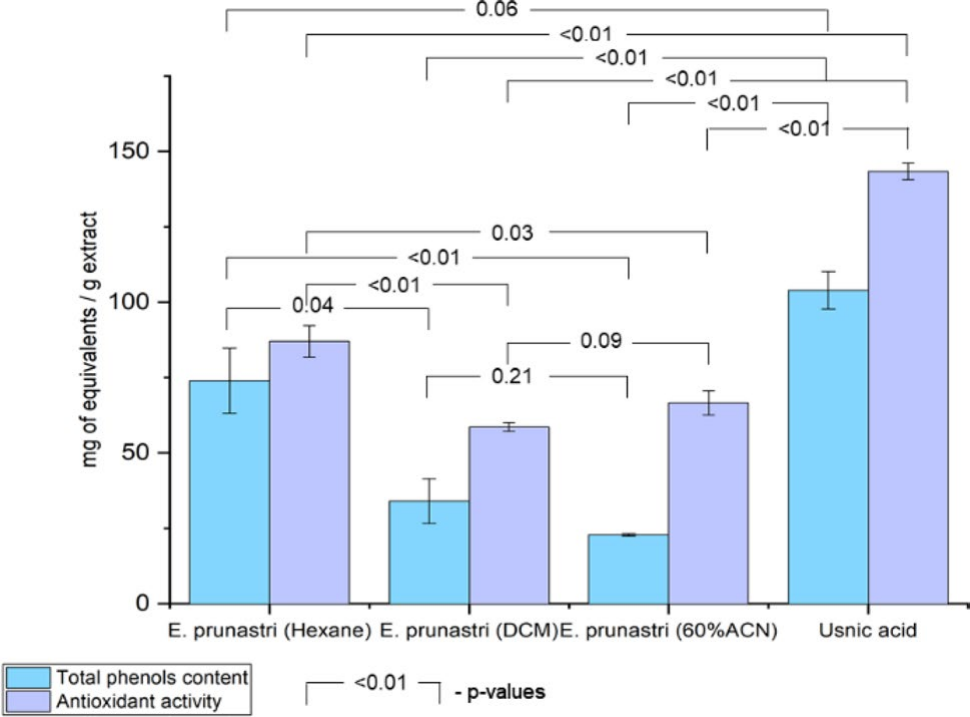
Antioxidant activity and total phenolic content of the *E. prunastri* extracts and usnic acid: total phenols, equivalents of gallic acid/g extract; antioxidant activity, mg ascorbic acid/g extract. Data are expressed as a mean ± SD (n = 3 for each experiment)

The antioxidant activity and the total phenol content of the different *E. prunastri* extracts in comparison to usnic acid are presented in Fig. 4. Usnic acid was used as a reference as it is a well-known lichen compound and is present in *E. prunastri*.

Usnic acid had shown the highest antioxidant activity (Fig. 4). Among the *E. prunastri* extracts, the hexane extract showed the highest antioxidant activity equivalent to 87 mg ascorbic acid per 1 g of extract (p < 0.01 and p = 0.03 vs. DCM and ACN extracts). The ACN and DCM extracts showed significantly lower activity equivalent to 67 and 59 mg ascorbic acid per 1 g of extract, respectively. These effects largely correspond with the total phenolic content: 74, 34 and 23 mg equivalent of gallic acid per 1 g of extract for Hexane, DCM and ACN extracts, respectively (p = 0.04 and p < 0.01 Hexane vs. DCM and ACN extracts). The difference between the DCM extract and the ACN extract is not significant (p = 0.09 for antioxidant activity and p = 0.21 for total phenols content). The ability of the extracts to deoxidize correlates directly to their total content of phenols (r = 0.82).

## Discussion

Usnic acid has been reported to be effective against antibiotic-resistant Gram-positive bacteria, e.g. vancomycin-resistant enterococci and methicillin-resistant *S. aureus* (MRSA) (Araújo et al. 2015). Interestingly, the inhibitory activity of usnic acid was lower against antibiotic susceptible strains of *S. aureus* and higher against MRSA strains (Tozatti et al. 2016; Victor et al. 2018; Sinha et al. 2019). The MIC values of usnic acid against different clinical isolates of *S. aureus* vary between 8 and 50 µg/ml (Gupta et al. 2012; Pompilio et al. 2013, 2016; Maciąg-Dorszyńska et al. 2014; Tozatti et al. 2016). In general, our results are corresponding to published ones.

There is less information about the antimicrobial activity of *E. prunastri*. An acetone extract from *E. prunastri* has been published to be active against *S. aureus* at the concentration range of 0.078–0.5 mg/ml and a MRSA clinical isolate at the concentration of 0.039 mg/ml (Tapalsky et al. 2017; Aoussar et al. 2020). A methanol extract was active at the concentration of 0.156 mg/ml (Mitrović et al. 2011). Results obtained in this research demonstrated much higher activity. In the case of *E. prunastri* Hexane extract, the effect on *S. aureus* was similar to the one of usnic acid. This broad MIC range could be due to different experiment setups, such as the number of bacteria used since the higher MIC was observed when a higher number of colony-forming units (CFU) was utilizing than for the lower MIC. The quantity of seeded microorganisms has been argued to thus effect microdilution MIC outcome (Strömstedt et al. 2017).

Against *P. aeruginosa,* usnic acid demonstrated a low antibacterial activity but lay within the broad range of earlier reported for usnic acid MIC values of 5.2 and 256 µg/ml (Francolini et al. 2004; Victor et al. 2018). The 50-fold higher MIC was observed when a 50-times higher number of colony-forming units (CFU) was utilizing than for the lower MIC. Different extracts from *E. prunastri* have been published to be active against *P. aeruginosa* at concentrations of 2.5–25 mg/ml (Mitrović et al. 2011; Aoussar et al. 2020). Another publication regarding to acetone extract from *E. prunastri* described no effect on *P. aeruginosa* at a concentration below 500 µg/ml (Tapalsky et al. 2017). In our case activity of the extracts against *P. aeruginosa* was comparable to the one of usnic acid, but higher than the published one.

Against another Gram-negative bacteria *E. coli*, usnic acid is reported to be effective with MIC values ranging from 5.2 to 20 µg/ml (Maciąg-Dorszyńska et al. 2014; Victor et al. 2018) for the different sensitive strains and more than 100 µg/ml for resistant strains of *E. coli* (Victor et al. 2018). Again, the difference in results is likely due to the different concentrations of bacteria used. In the current study, 10^6^ CFU/ml was used whereas Victor et al. used a concentration of 10^4^ CFU/ml. Comparing to those, our investigations showed usnic acid as less effective. The activity of the extracts was comparable to one of usnic acid and corresponding to the published results (Tapalsky et al. 2017).

The activity of usnic acid against *C. albicans* was much lower than a reported one with the MIC of 75 µg/ml (Nithyanand et al. 2015). In contrast to this, the extracts showed higher activity than a published one (Tapalsky et al. 2017), but with MIC in the range of usnic acid. The only difference was found for the *E. prunastri* ACN, which was significantly more active than usnic acid.

Additionally, having a phenolic nature, usnic acid possesses also antioxidant capacities (Araújo et al. 2015). It is known, that phenols have a high antioxidant capacity, mostly due to their reduction ability (Garcia-Mateos 2017). They are able to capture and neutralize free radicals and reactive oxygen species (Wei et al. 2012). Extracts from *E. prunastri*, in the turn, showed moderate antioxidant activity comparing to usnic acid and a lower number of phenols. Obtained results are corresponding to the published ones (Mitrović et al. 2011; Hawrył et al. 2020; Aoussar et al. 2020).

We demonstrated that *E. prunastri* extracts possess antioxidant and antimicrobial activities. Difference in activities are based on different compound compositions of the extracts and it was identified for the first time in this study that the hexane extract yields the highest antioxidative activity whereas the ACN extract possesses the highest antimicrobial activity against all tested microorganisms. The MIC values of the ACN extract were comparable to usnic acid, i.e., 21, 133, 225 and 100 µg/ml against *S. aureus, P. aeruginosa, E. coli and C. albicans,* respectively.

The bioassay-guided fractionation of *E. prunastri* ACN extract showed that some fractions obtained had higher activity. Especially, fractions V and VII showed activities comparable to published data on usnic acid (Gupta et al. 2012; Pompilio et al. 2013, 2016; Maciąg-Dorszyńska et al. 2014; Tozatti et al. 2016).

To put obtained results into perspective, the Clinical and Laboratory Standards Institute (CLSI) recommends MIC values from 0.12 to 32 μg/ml as effective concentrations against *S. aureus* for conventional antibiotics depending on the class (CLSI 2018). The European Committee on Antimicrobial Susceptibility Testing (EUCAST) describes the effective concentrations against *S. aureus* in the MIC range from 0.03 to 16 µg/ml for different classes of antibiotics (EUCAST 2019). Thus, in the case of our study *S. aureus* may be considered susceptible to fractions V to X and XIV.

The effect of those fractions on Gram-negative bacteria *P. aeruginosa* and *E. coli* was low. Both bacteria can be considered resistant to these fractions according to the CLSI and EUCAST recommendations (CLSI 2018; EUCAST 2019).

Against *C. albicans,* fractions V to VIII showed effect better than usnic acid, and, despite this, according to the EUCAST, *C. albicans* is non-susceptible to the tested fractions (EUCAST 2020).

Fraction VI showed the highest inhibitory effects against all tested microorganisms in concentrations usually observed for intermediately potent antibiotics (4–25 µg/ml). In this fraction, the most abundant compound was identified as evernic acid.

The chemical characterization of the total extract of *E. prunastri* earlier (Staples et al. 2020) showed that usnic acid, lecanoric acid and chloroatranorin were among the compounds produced in addition to evernic acid at high concentrations, with evernic acid being the most abundant compound (Staples et al. 2020). Besides chloroatranorin also atranorin was present but in smaller amounts. It may be interesting to note that authors, when comparing *E. prunastri* lichens from three different geographical regions, could detect two compounds — the depsidones salazinic acid and physodic acid — only in *E. prunastri* collected in Mari-El, Russian Federation that is used in the current study.

Data on mechanisms of the antimicrobial action of evernic acid are not available. Only data on antibiofilm and antibiofilm maturation activity of an *E. prunastri* extract against *C. albicans* (Millot et al. 2017), as well as inhibiting quorum sensing activity against *P. aeruginosa* (Gökalsın and Sesal 2016) has been identified. The mechanism of action against *P. aeruginosa* quorum sensing is based on the inhibition of *lasB* and *rhlA* genes expression (Gökalsın and Sesal 2016).

Staples et al. demonstrated earlier that evernic acid is the main constituent of *E. prunastri* (Staples et al. 2020). Considering our findings and the ones from the biofilm experiments (Gökalsın and Sesal 2016) it can be hypothesized that evernic acid is one of the main constituents responsible for the inhibition of the biofilm formation (especially for *P. aeruginosa* and *C. albicans*).

However, some mechanisms of antimicrobial activity for usnic acid are described. Against Gram-positive bacteria *S. aureus*, usnic acid can act as a membrane-damaging agent (Gupta et al. 2012). Moreover, investigations on *Bacillus subtilis* in models of artificial planar bilayer lipid membranes and rat liver mitochondria have demonstrated that a potential mechanism of the antimicrobial capacities of usnic acid can be related to its protonophoric activity (Antonenko et al. 2019). Being lipophilic, usnic acid permeates the bilayer membrane, causing proton leakage, and disrupts the bacterial membrane (Lauinger et al. 2013; Antonenko et al. 2019). Evernic acid, also being lipophilic (ChEMBL, https://www.ebi.ac.uk/chembl/) with a slight structural similarity to usnic acid, may — hypothetically — also be able to compromise bacterial cell membranes and thereby exert antimicrobial activity.

The influence of usnic acid on a prooxidant-antioxidant balance has been described as a mechanism of the anti-candida effect (Peralta et al. 2017). The antioxidant properties of the extract containing evernic acid are significantly lower compared to usnic acid. Thus, the anti-candida mechanism of evernic acid is not necessarily related to its antioxidant properties.

Another possible mechanism of usnic acid activity against *Mycobacterium tuberculosis, E. coli, S. aureus* is the binding to allosteric sites on the FabI (enoyl reductase enzyme) protein surface, which affects enzyme activity (Lauinger et al. 2013). Moreover, this mechanism is described for the activity of usnic acid against the blood-stage *Plasmodium falciparum* as well as the liver stage *P. berghei* (Lauinger et al. 2013). At the same time, evernic acid inhibited FabI and FabZ of *Plasmodium falciparum* and FabI of *E. coli* and *S. aureus* (Lauinger et al. 2013)*.* Additionally, usnic acid shows activity against other parasites of *Leishmania* spp. by inducing apoptosis (Derici et al. 2018).

Atranorin has been described to be active against Gram-negative bacteria which is uncommon for other known lichen compounds (Studzinska-Sroka et al. 2017). The presence of atranorin or (and) structural similarity of evernic acid could thus explain the activity against Gram-negative bacteria observed in our experiments.

Also, evernic acid is described to downregulate the expression of the genes lasB (elastase) and rhlA (rhamnolipid production) which are virulence factors in *P. aeruginosa* (Gökalsın and Sesal 2016)*.*

The scaffold of evernic acid is phenyl salicylate (ZINC, http://zinc.docking.org/). Salicylates have been shown to possess antibiofilm and anti-metabolic effects on bacteria (Damman 2013).

Despite usnic acid has been shown to be hepatotoxic (Guo et al. 2008; Luzina and Salakhutdinov 2018) the toxicity of other lichen compounds have been barely investigated to date. With the support of in silico modelling, usnic acid and depsidones were predicted to have toxic risks, corresponding to the publications. On the other hand, depsides (a class of evernic acid) were not associated with the toxicity risks.

It is interesting to note, that a whole methanol *E. prunastri* extract has been screened for its antigenotoxic capacity against several mutagens such as *N*-methyl-*N*′-nitro-*N*-nitrosoguanidine, acridin and aflatoxin using WP2, Ames and sister chromatid exchange test systems (Alpsoy et al. 2015). The data obtained have clearly shown that the extract has significant antigenotoxic effects which are thought to be partly due to the antioxidant activities and antioxidant inducing capability (Alpsoy et al. 2015).

Numbers of research describe lichen compounds as potential antibiotics (especially against Gram-positive bacteria) (Shrestha and Clair 2013; Francolini et al. 2019; Sepahvand et al. 2021). To date, the lichen compounds were not ascribed to any antibiotic class. A structural similarity comparison of evernic acid revealed no structural similarity with such antibiotic classes as Cell wall synthesis inhibitors and Protein synthesis inhibitors. Evernic acid and other depsides have structures slightly similar to Folic acid synthesis inhibitors and DNA synthesis inhibitors.

Folate (folic acid) is a designation for cofactors that consist of three moieties: *para*-aminobenzoic acid (PABA), pterin and glutamates (Bertacine Dias et al. 2018). Tetrahydrofolate (a pterin ring) has an essential role in the biosynthesis of purines, thymidylate, pantothenate, RNA and amino acids (Bertacine Dias et al. 2018). Among folic acid synthesis inhibitors, sulfamethoxazole and trimethoprim were the most similar to evernic acid compounds. Sulfamethoxazole being similar to PABA compete with the latter during synthesis of dihydrofolate (Kemnic and Coleman 2021). Trimethoprim is also a dihydrofolate inhibitor. Alternatively to sulfamethoxazole, it halts the production of tetrahydrofolate, which is necessary for the biosynthesis of bacterial nucleic acids and proteins (Kemnic and Coleman 2021).

DNA synthesis inhibitors (quinolones) block DNA replication apparatus via the binding to the complexes of DNA with DNA topoisomerase IV and DNA gyrase (Hooper and Jacoby 2016). The DNA topoisomerase IV and DNA gyrase catalyse double-strand break followed by duplication of the strands (Hooper and Jacoby 2016). Fluoroquinolones are quinolones substituted with fluorine. Nalidixic acid and norfloxacin are fluoroquinolones are the most similar to evernic acid. Moxifloxacin and gatifloxacin were found the most identical to usnic acid. All described antibiotics act as DNA gyrase inhibitors (Mohammed et al. 2019).

This was also the case for other metabolites of *E. prunastri* as atranorin, chloratranorin, lecanoric acid and the two depsidones salazinic acid and physodic acid. These compounds are structurally similar to evernic acid and are therefore possible candidates for antibiotics representing a novel class of substances with antimicrobial activity.

## Supplementary Information

Below is the link to the electronic supplementary material.

Supplementary file1 (PDF 42 kb)

Supplementary file2 (DOCX 18 kb)

## Data Availability

Not applicable.
