# Supplementary material for: Antimicrobial and antioxidant activity of Evernia prunastri extracts and their isolates
Source: World J Microbiol Biotechnol. 2021 Jul 7;37(8):129. doi: 10.1007/s11274-021-03099-y (PMC8263414; doi:10.1007/s11274-021-03099-y)
Supplement: Supplementary file 1 — Supplementary file1 (PDF 42 kb) [file 11274_2021_3099_MOESM1_ESM.pdf]

The yield of different fractions obtained in silica gel column chromatography during the separation of 60% ACN *E. prunastri* extract

Batch	Fraction	Yield, mg
Eluent: Cyclohexane : acetone / 7:4		
I	1-6	5.0
II	7-15	3.4
III	16-26	12.4
IV	27-36	10.7
V	37-50	10.6
Eluent: Cyclohexane : acetone : methanol / 7:4:1		
VI	51-68	31.8
VII	69-79	31.1
VIII	80-82	44.9
IX	83-84	12.6
Eluent: Cyclohexane: acetone : methanol / 7:4:2		
X	85	0.7
X (E.pr IV)	85	0.7
XI (E.pr V)	86	0.7
XII (E.pr VI)	87	5.0
XIII (E.pr VII)	88	5.8
Eluent: Methanol		
XIV	90	11.8
XV	91	224.6
