# Supplementary material for: Antimicrobial and antioxidant activity of Evernia prunastri extracts and their isolates
Source: World J Microbiol Biotechnol. 2021 Jul 7;37(8):129. doi: 10.1007/s11274-021-03099-y (PMC8263414; doi:10.1007/s11274-021-03099-y)
Supplement: Supplementary file 2 — Supplementary file2 (DOCX 18 kb) [file 11274_2021_3099_MOESM2_ESM.docx]

Structural similarity of evernic acid and usnic acid to known antibiotics calculated with DataWarrior software based on the comparison of descriptors – FragFp

| **Name** | **Mechanism** | **Similarity to evernic acid (FragFp)** | **Similarity to usnic acid (FragFp)** |
| --- | --- | --- | --- |
| Penicillin G | Cell Wall Synthesis Inhibitors (Penicillins) | 0.087193 | 0.34307 |
| Penicillin V | Cell Wall Synthesis Inhibitors (Penicillins) | 0.10193 | 0.34223 |
| Ampicillin | Cell Wall Synthesis Inhibitors (Penicillins) | 0.088 | 0.34856 |
| Amoxicillin | Cell Wall Synthesis Inhibitors (Penicillins) | 0.10554 | 0.3717 |
| Methicillin | Cell Wall Synthesis Inhibitors (Penicillins) | 0.16578 | 0.41247 |
| Nafcillin | Cell Wall Synthesis Inhibitors (Penicillins) | 0.18085 | 0.42823 |
| Oxacillin | Cell Wall Synthesis Inhibitors (Penicillins) | 0.12183 | 0.38695 |
| Cloxacillin | Cell Wall Synthesis Inhibitors (Penicillins) | 0.13784 | 0.40509 |
| Dicloxacillin | Cell Wall Synthesis Inhibitors (Penicillins) | 0.14536 | 0.41204 |
| Carbenicillin | Cell Wall Synthesis Inhibitors (Penicillins) | 0.084881 | 0.36077 |
| Ticarcillin | Cell Wall Synthesis Inhibitors (Penicillins) | 0.092593 | 0.35407 |
| Piperacillin | Cell Wall Synthesis Inhibitors (Penicillins) | 0.10209 | 0.35059 |
| Cefazolin | Cell Wall Synthesis Inhibitors (Cephalosporins) | 0.11573 | 0.3225 |
| Cephalexin | Cell Wall Synthesis Inhibitors (Cephalosporins) | 0.12392 | 0.34406 |
| Cefoxitin | Cell Wall Synthesis Inhibitors (Cephalosporins) | 0.12079 | 0.35294 |
| Cefaclor | Cell Wall Synthesis Inhibitors (Cephalosporins) | 0.12607 | 0.34483 |
| Cefuroxime | Cell Wall Synthesis Inhibitors (Cephalosporins) | 0.12684 | 0.34085 |
| Ceftriaxone | Cell Wall Synthesis Inhibitors (Cephalosporins) | 0.12048 | 0.32576 |
| Cefotaxime | Cell Wall Synthesis Inhibitors (Cephalosporins) | 0.1276 | 0.33584 |
| Ceftazidime | Cell Wall Synthesis Inhibitors (Cephalosporins) | 0.125 | 0.36098 |
| Cefepime | Cell Wall Synthesis Inhibitors (Cephalosporins) | 0.10888 | 0.32678 |
| Vancomycin | Cell Wall Synthesis Inhibitors (Vancomycin) | 0.21077 | 0.54023 |
| Clavulanic Acid | Cell Wall Synthesis Inhibitors (beta-lactamase inhibitors) | 0.11974 | 0.3414 |
| Sulbactam | Cell Wall Synthesis Inhibitors (beta-lactamase inhibitors) | 0.073314 | 0.31726 |
| Tazobactam | Cell Wall Synthesis Inhibitors (beta-lactamase inhibitors) | 0.09434 | 0.34053 |
| Imipenem | Cell Wall Synthesis Inhibitors (Carbapenems) | 0.10773 | 0.39196 |
| Cilastatin | Cell Wall Synthesis Inhibitors (Carbapenems) | 0.13571 | 0.37318 |
| Meropenem | Cell Wall Synthesis Inhibitors (Carbapenems) | 0.10183 | 0.3789 |
| Doripenem | Cell Wall Synthesis Inhibitors (Carbapenems) | 0.1013 | 0.37709 |
| Ertapenem | Cell Wall Synthesis Inhibitors (Carbapenems) | 0.14394 | 0.42254 |
| Aztreonam | Cell Wall Synthesis Inhibitors (Aztreonam) | 0.11527 | 0.36364 |
| Bacitracin | Cell Wall Synthesis Inhibitors (Bacitracin) | 0.10188 | 0.40648 |
| Gentamicin | Protein Synthesis Inhibitors (Aminoglycosides) | 0.055118 | 0.22029 |
| Neomycin | Protein Synthesis Inhibitors (Aminoglycosides) | 0.059322 | 0.1994 |
| Amikacin | Protein Synthesis Inhibitors (Aminoglycosides) | 0.073718 | 0.30481 |
| Tobramycin | Protein Synthesis Inhibitors (Aminoglycosides) | 0.060345 | 0.1982 |
| Streptomycin | Protein Synthesis Inhibitors (Aminoglycosides) | 0.073248 | 0.32075 |
| Tetracycline | Protein Synthesis Inhibitors (Tetracyclines) | 0.1631 | 0.48861 |
| Doxycycline | Protein Synthesis Inhibitors (Tetracyclines) | 0.15467 | 0.49109 |
| Minocycline | Protein Synthesis Inhibitors (Tetracyclines) | 0.1811 | 0.50374 |
| Demeclocycline | Protein Synthesis Inhibitors (Tetracyclines) | 0.17241 | 0.49874 |
| Erythromycin | Protein Synthesis Inhibitors (Macrolides) | 0.068966 | 0.35659 |
| Azithromycin | Protein Synthesis Inhibitors (Macrolides) | 0.066298 | 0.34414 |
| Clarithromycin | Protein Synthesis Inhibitors (Macrolides) | 0.067989 | 0.35897 |
| Chloramphenicol | Protein Synthesis Inhibitors (Chloramphenicol) | 0.14981 | 0.31054 |
| Clindamycin | Protein Synthesis Inhibitors (Lincosamide) | 0.068182 | 0.30941 |
| Linezolid | Protein Synthesis Inhibitors (Linezolid) | 0.18121 | 0.41854 |
| Quinupristin | Protein Synthesis Inhibitors (Streptogramins) | 0.14286 | 0.40044 |
| Dalfopristin | Protein Synthesis Inhibitors (Streptogramins) | 0.10302 | 0.38318 |
| Nalidixic acid | DNA Synthesis Inhibitors (Fluoroquinolones) | 0.33523 | 0.37102 |
| Ciprofloxacin | DNA Synthesis Inhibitors (Fluoroquinolones) | 0.30802 | 0.44688 |
| Norfloxacin | DNA Synthesis Inhibitors (Fluoroquinolones) | 0.33486 | 0.46535 |
| Enoxacin | DNA Synthesis Inhibitors (Fluoroquinolones) | 0.29717 | 0.42193 |
| Ofloxacin | DNA Synthesis Inhibitors (Fluoroquinolones) | 0.31518 | 0.4924 |
| Levofloxacin | DNA Synthesis Inhibitors (Fluoroquinolones) | 0.31518 | 0.4924 |
| Gatifloxacin | DNA Synthesis Inhibitors (Fluoroquinolones) | 0.29368 | 0.49552 |
| Moxifloxacin | DNA Synthesis Inhibitors (Fluoroquinolones) | 0.24383 | 0.50678 |
| Gemifloxacin | DNA Synthesis Inhibitors (Fluoroquinolones) | 0.22184 | 0.48547 |
| Metronidazole | DNA Synthesis Inhibitors (Metronidazole) | 0.16 | 0.2623 |
| Rifampin | RNA Synthesis Inhibitors | 0.24776 | 0.51862 |
| Isoniazid | Mycolic Acid Synthesis Inhibitors | 0.23423 | 0.08209 |
| Trimethoprim | Folic Acid Synthesis Inhibitors | 0.34211 | 0.18605 |
| Sulfamethoxazole | Folic Acid Synthesis Inhibitors | 0.38129 | 0.20629 |
| Pyrimethamine | Folic Acid Synthesis Inhibitors | 0.19091 | 0.096525 |
| Evernic acid | Unknown | 1.00 | 0.28253 |
| Usnic acid | Unknown | 0.28253 | 1 |
| Lecanoric acid | Unknown | 0.99 | 0.28253 |
| Chloratranorin | Unknown | 0.87037 | 0.30515 |
| Salacinic acid | Unknown | 0.61039 | 0.37801 |
| Physodic acid | Unknown | 0.63158 | 0.4635 |
| Atranorin | Unknown | 0.88679 | 0.30741 |
